# Training Intensity Distribution and Changes in Performance and Physiology of a 2nd Place Finisher Team of the Race across America Over a 6 Month Preparation Period

**DOI:** 10.3389/fphys.2016.00642

**Published:** 2016-12-27

**Authors:** Christian Manunzio, Joachim Mester, Walter Kaiser, Patrick Wahl

**Affiliations:** ^1^Institute of Training Science and Sport Informatics, German Sport UniversityCologne, Germany; ^2^The German Research Centre of Elite Sport, German Sport University CologneCologne, Germany; ^3^Private Practice for Oral and Maxillofacial SurgeryNeuss, Germany; ^4^Department of Molecular and Cellular Sport Medicine, Institute of Cardiovascular Research and Sport Medicine, German Sport University CologneCologne, Germany

**Keywords:** critical power, VO_2max_, maximal rate of lactate accumulation, MLSS, lactate minimum intensity, ultra-endurance performance

## Abstract

**Aim:** To monitor the training intensity distribution (TID) and the development of physiological and performance parameters.

**Methods:** During their preparation period for the RAAM, 4 athletes (plus 1 additional backup racer) performed 3 testing sessions; one before, one after 3, and one after 6 months of training. VO_2max_, maximal rate of lactate accumulation (dLa/dt_max_), critical power, power output at lactate minimum (MLSS_P_), peak and mean power output during a sprint test, heart rate recovery, isometric strength, jumping height, and body composition were determined. All training sessions were recorded with a power meter. The endurance TID was analyzed based on the time in zone approach, according to a classical 3-zone model, including all power data of training sessions, and a power specific 3-zone model, where time with power output below 50% of MLSS_P_ was not considered.

**Results:** The TID using the classical 3-zone model reflected a pyramidal TID (zone 1: 63 ± 16, zone 2: 28 ± 13 and zone 3: 9 ± 4%). The power specific 3-zone model resulted in a threshold-based TID (zone 1: 48 ± 13, zone 2: 39 ± 10, zone 3: 13 ± 4%). VO_2max_ increased by 7.1 ± 5.3% (*P* = 0.06). dLa/dt_max_ decreased by 16.3 ± 8.1% (*P* = 0.03). Power output at lactate minimum and critical power increased by 10.3 ± 4.1 and 16.8 ± 6.2% (*P* = 0.01), respectively. No changes were found for strength parameters and jumps.

**Conclusion:** The present study underlines that a threshold oriented TID results in only moderate increases in physiological parameters. The amount of training below 50% of MLSSp (~28% of total training time) is remarkably high. Researchers, trainers, and athletes should pay attention to the different ways of interpreting training power data, to gain realistic insights into the TID and the corresponding improvements in performance and physiological parameters.

## Introduction

The Race Across America (RAAM) is an annual 4800 km non-stop cycling race from the west coast of America to the east coast. The physiological and environmental challenges encountered by the athletes participating in this event are numerous: insufficient energy intake (Knechtle et al., 2005; Hulton et al., 2010), sleep deprivation (Hulton et al., 2010; Lahart et al., 2013) and tough climate conditions like extreme heat in the desert or very high humidity resulting in a decline in performance due to dehydration (Bowen et al., 2006; Paulin et al., 2015). Additionally, over 30,000 m of altitude difference and the corresponding time under hypoxic conditions make the event one of the toughest bike races in the world and the unofficial world championship of ultra-endurance cycling. The race can be performed as a team event, with 2, 4, or 8 racers as a relay team or solo.

The performance requirements for a team relay in the RAAM can be described as an enormous number of individual time trials, interspersed by longer and shorter phases of recovery, depending on the team tactics. The physiological load can, therefore, be compared to the bike split in triathlon events, where performance (after preload by the swim split) is ideally as high as possible without impeding the following run split. Under ideal conditions, each team member in a 4 person relay team would have to cover 1200 km on the bike as fast as possible.

Concerning the physiological prerequisites, Laursen et al. (1999) described a RAAM relay team with 4 racers, finishing in fourth place with a mean maximal oxygen uptake (VO_2max_) of 71.5 mL·min^−1^·kg^−1^,while a solo racer winning the RAAM was described by Ice et al. (1988) with a VO_2max_ of 79.6 mL·min^−1^·kg^−1^. These values correspond roughly to the range of VO_2max_ values for professional cyclists from 69.7 mL·min^−1^·kg^−1^ to 84.8 mL·min^−1^·kg^−1^summarized by Padilla et al. (1999). However, Faria et al. (2005) showed in their review of cycling physiology, that VO_2max_ alone is neither a good predictor of endurance performance nor a valid distinction between elite and amateur cyclists. Lactate threshold (LT) might be more suitable as a predictor of endurance performance since it was the single variable that correlated best with time trial performance (*r* = 0.86, *p* = 0.01) in the study of Støren et al. (2013). This might be associated with the findings of Jacobs et al. (2011), who demonstrated that endurance performance among highly trained cyclists correlated with skeletal muscle oxidative capacity, accounting for 47% of the variation in 26 km time trial performance. However, no previous study did report comprehensively on the development of aerobic [e.g., VO_2max_, maximal lactate steady state (MLSS)] and anaerobic parameters [e.g., maximal rate of lactate accumulation (dLa/dt_max_), maximal voluntary contraction (MVC)] in combination with performance measures [peak power output (PPO), sprint PPO, sprint mean power output (MPO), critical power (CP)] over the course of a preparation period for an ultra-endurance cycling race.

On the topic of training to optimize performance for such extreme endurance challenges, different information can be found in scientific literature. Stöggl and Sperlich (2015) proposed different concepts of training intensity and volume distribution (TID) in the physical conditioning of endurance athletes: (1) high-volume low-intensity training (HVLIT), (2) training at or near the lactate threshold (THR), (3) low-volume high-intensity interval training (HIT), (4) “pyramidal” (PYR) TID consisting of high volume of HVLIT, medium volume of THR, and small volume of HIT, (5) as well as the combination of HVLIT and HIT, named “polarized” training (POL).

There are a number of prospective and intervention studies in cycling (Neal et al., 2013), running (Esteve-Lanao et al., 2007; Muñoz et al., 2014), different endurance disciplines (Stöggl and Sperlich, 2014), as well as retrospective studies in speed skating (Yu et al., 2012), running (Billat et al., 2001), and cross-country skiing (Seiler and Kjerland, 2006), reporting the superiority of a POL TID compared to other TIDs.

However, there are also many retrospective studies, generally reporting a PYR TID for endurance athletes in running (Esteve-Lanao et al., 2005), cycling (Luciá et al., 2000; Schumacher and Mueller, 2002; Zapico et al., 2007), and triathlon (Neal et al., 2011). In a recent review, it was summarized that most retrospective studies on well-trained athletes report on PYR TID, although some world-class athletes performed POL TID during certain phases of the season (Stöggl and Sperlich, 2015). Sandbakk et al. (2016), Tønnessen et al. (2014), and Guellich et al. (2009) reported a general focus on HVLIT TID in the preparation period, which became more polarized in the competition period of world/national class cross-country skiers, biathletes, and young world-class rowers.

Most studies reporting TID used the heart rate (HR) or the rate of perceived exertion (RPE) to determine training time in different intensity zones. To the best of our knowledge, there are no studies using power output for the determination of TID. The ability to accurately quantify the mechanical work during training makes cycling unique in allowing such insights into the demands of sporting preparation. Power output as a marker of the external load is zero in phases like downhill sections or during coasting, but the internal load represented by the HR might still appear higher (Halson, 2014), resulting in a “smoothing effect.” Especially very short high-intensity accelerations (e.g., initial pedal strokes or short intervals), as well as abrupt reduction of external load, will not be reflected by the HR. Additionally, Seiler and Kjerland (2006) demonstrated very big differences in TID between the total time-in-zone approach and the session-goal method. Many factors may influence the relationship between workload and HR (Buchheit, 2014), and day-to-day variation in HR was shown to be approximately 6.5%, which might influence the analysis of HR data and consequently the time spent in each zone (Bagger et al., 2003). However, Davies and Knibbs (1971) found, that an intensity of at least 50% of VO_2max_ is necessary to result in performance enhancements. This raises the question how TID (time in each zone in a 3 zone-model) based on power data might change: 1. if all power data of training are included in the analysis and are assigned to one of the 3 zones, or 2. if power data below a certain threshold are excluded from the analysis.

We accompanied 6 months of preparation time of a 4 person RAAM team (plus 1 additional backup racer). They later finished the actual race in second place. All athletes were amateurs, and their training time was restricted by social and environmental conditions. The first aim of this study was to add a more detailed physiological and performance profile for long-distance cyclists over the course of their preparation period, including not only aerobic (VO_2max_, MLSS, CP, PPO), but also anaerobic (dLa/dt_max_, sprint PPO, MPO, MVC) measurements. The second aim was to analyze which of the physiological variables (VO_2max_, MLSS, dLa/dt_max_) might correlate with performance (CP, sprint MPO), including the values of all three testing sessions, in order to see which of the laboratory parameters might be useful to predict performance. The third aim was to examine the composition of the training load based on power data with regards to TID. More specifically, changes in the TID are compared in two conditions: when all power data are included, or when power data below a certain threshold are eliminated.

## Methods

### Subjects

Four male experienced cyclists and triathletes (more than 15 years of regular endurance training) and one backup athlete (45 ± 6.5 years; 182.2 ± 8.9 cm; 79.5 ± 6.6 kg) in preparation for the RAAM volunteered to participate in this study over 6 months. The preparation period started after the offseason. During that offseason athletes had a mean training time of ~8 h per week, consisting of cycling, running and swimming. The study protocol was approved by the University's ethics review board and is in accordance with the declaration of Helsinki. Each subject gave it's written informed consent and was informed about possible risks of participation.

### Design

Each athlete underwent three single day testing sessions: The first at the beginning of the preparation period, the second after 3 months of training (phase 1) and the third at the end of the 6 months training period (phase 2), 2 weeks before the race. Each diagnostic session was completed in the same manner at the same time of day. Prior to performance testing, the subject's body mass and lean body mass were measured using a four-electrode bioimpedance body scale (BC 418 MA, Tanita Corp., Tokyo, Japan). Performance testing consisted of jump and strength tests, followed by endurance tests on a cycle ergometer. Subjects were instructed to arrive in the laboratory in a rested, 2 h postprandial and fully hydrated state. They were ordered to avert strenuous exercise for at least 24 h before each test. The order of the tests was kept identical for each individual in the following order. Between the different tests, adequate resting time was ensured.

### Squat jump (SJ) and counter movement jump test (CMJ)

For the SJ, the subjects were instructed to place the hands on the hips and to lower the hip into a squat position with a knee angle of 90°. Out of this position they had to jump with both legs for maximal height. For the CMJ, the subjects were instructed to place the hands on the hips and to lower the hip dynamically down to a self-selected level before jumping with both legs for maximal height. Hands remained on the hips for the entire movement in both tests to eliminate any influence of the arm swing. Flight time was measured using the Optojump (Microgate Srl, Italy) system, which calculated the jump height. Subjects performed three SJ and three CMJ and the maximal jump height was taken for later analysis (Brown and Weir, 2001).

### Strength-tests

Maximal voluntary isometric strength (MVC) was tested with both legs on a leg press (LP), a leg extension (LE), and a leg curl (LC) machine (Edition-Line, gym80, Gelsenkirchen, Germany), which were equipped with the digital measurement technique Digimax (mechaTronic; Hamm, Germany) to make measurements of force-time and velocity-time variables (5 kN strength sensor type KM1506, distance sensor type S501D, megaTron; Munich, Germany) with the included software IsoTest and DynamicTest 2.0, as described previously (Wahl et al., 2016). The sensors were installed in line with the steel band of the machines that lifts the weight plates. Maximum force relative to body weight was calculated for statistical analysis and data presentation. Subjects performed three trials of each test, and the maximal value was taken for later analysis.

### Cycling-tests

All tests were performed on an SRM Ergometer (Schoberer Radmesstechnik, GmbH, Germany, Jülich), with seat and handlebar height kept identical for each subject throughout all tests.

### Sprint-test

After an initial warm-up at 2 W·kg^−1^ for 10 min, followed by 5 min of passive rest, a 15 s all-out sprint test was performed in an isokinetic mode set to a cadence of 120 rpm. The subjects performed the test in a sitting position on the ergometer and were verbally encouraged to achieve maximal power output throughout the test. Afterward, peak power (sprint PPO) and mean power (sprint MPO) were determined. Capillary samples from the earlobe were collected before and in minute intervals (1′–10′) after the test to determine the “maximal rate of lactate accumulation (dLa/dt_max_)” according to Heck et al. (2003) and Hauser et al. (2014a):

$$\begin{array}{l} dLa/dt_{max}\;\left(mmol\cdot L^{-1}\cdot s^{-1}\right)=\left({\left[La\right]}_{max}-{\left[La\right]}_{rest}\right)\;{\left(t_{exerc}-t_{alac}\right)}^{-1} \end{array}$$

where [*La*]_*max*_ (*mmol*·*L*^−1^) = maximal lactate concentration after the exercise; [*La*]_*rest*_ (*mmol*·*L*^−1^) = lactate concentration before exercise; *t*_*exerc*_ (seconds) = duration of exercise; *t*_*alac*_ (seconds) = period at the beginning of exercise for which (fictitiously) no lactate formation is assumed. The *t*_*alac*_ for each subject was set as the time to sprint PPO (seconds).

### VO_2max_-test

After the sprint test and additional 10 min of recovery, the athletes performed a maximal incremental exercise test (initial load 100 W + 20 W 1 min^−1^) until exhaustion to determine the VO_2max_ and the corresponding workload (PPO). Heart rate (Polar, Kempele, Finland), VO_2_ and carbon dioxide output (VCO_2_) (Cortex Metalyzer II, Leipzig, Germany) were continuously measured during the test.

Afterward, the VO_2max_ and dLa/d_tmax_ were used to calculate the maximal lactate steady state (MLSS_c_) and the lactate turn point 1 (LT1) according to Hauser et al. (2014a).

### Lactate-minimum-test (LMT)

A modified lactate minimum test (Knöpfli-Lenzin and Boutellier, 2011) was performed exactly 7 min after finishing the VO_2max_ test. After these 7 min of passive recovery, RPE, lactate and heart rate were measured. The drop in heart rate within this time was used to calculate a heart rate recovery index (HRR). Subjects started a second incremental test, beginning with a workload 50 W below the MLSS_c_. The workload was increased by 10 w every 90 s, until complete exhaustion. Heart rate (Polar, Kempele, Finland) was continuously measured during the test, RPE and lactate samples were obtained out of the earlobe at the end of each step. Power output at the step which elicited lactate minimum (LM) was considered as maximal lactate steady state power (MLSS_P_), according to Knöpfli-Lenzin and Boutellier (2011).

### Short power-profile

Besides the laboratory measurements, the athletes underwent 3 mean maximal power (MMP) tests in the field over the duration of 2, 5, and 10 min. The MMP tests were each done on different days around the laboratory measurements. The results were used to calculate critical power (CP) according to Monod and Scherrer (1965).

### Training data

Training mainly consisted of endurance training on the bike. Additionally, athletes carried out individual core stability training, which was not recorded. Endurance training data of the subjects were captured using wireless SRM Cranks (Schoberer Radmesstechnik, GmbH, Germany, Jülich) and the Powercontrol 8 head unit (Schoberer Radmesstechnik, GmbH, Germany, Jülich), saving power data in 1-s intervals for later analysis.

The aggregation of the raw data was done in SRM Win 6.42.18 (Schoberer Radmesstechnik, GmbH, Germany, Jülich), further analysis was performed in Microsoft Excel 2010 (Microsoft Corporation, Redmond, USA) and Statistica 7.1 (StatSoft Inc., Tulsa, USA).

Power data were retrospectively analyzed and were used to determine the percentage of training time spent in each of three training zones for each individual training session.

The classical 3-zone model was defined as follows: zone 1 (below the calculated first rise of lactate/LT1, Hauser et al., 2014a), zone 2 (between LT1 and MLSS_P_), and zone 3 (above MLSS_P_). The average training time in each zone for all sessions of each subject was then determined according to Seiler and Kjerland (2006). Additionally, a power specific 3-zone model was defined by deleting all time with power output below 50% of MLSS_P_, in order to reduce the impact of coasting phases and similar events on the TID: zone 1 (between 50% of MLSS_P_ and LT1, Hauser et al., 2014a), zone 2 (between LT1 and MLSS_P_) and zone 3 (above MLSS_P_). Training zones were adjusted after each testing session according to the changes in LT1 and MLSS_P_ (Table 1). Individual race data were retrospectively analyzed for each of the 4 racers (time, distance, altitude difference, average relative power output, average power output, average power output in percent of MLSS_P_, average power output in percent of LT1, and average cadence).

**Table 1:** **Changes of power based training zones over the course of the preparation period for RAAM**.

**Test Nr**.	**1**	**2**	**3**
Zone 1	0/136^*^ ± 24–190 ± 42	0/141^*^ ± 17–197 ± 37	0/146^*^ ± 25–215 ± 42
Zone 2	191 ± 51–272 ± 48	198 ± 41–282 ± 34	216 ± 51–292 ± 50
Zone 3	>273 ± 48	>283 ± 34	>293 ± 50

* *lower limit of zone 1 for the power specific 3-zone model*.

### Statistical analysis

For all statistical analysis of the data Statistica (Version 7.1, StatSoft Inc., USA) software package for Windows® was used. Descriptive statistics of the data are presented as means ± standard deviation (± SD). Data were tested via skewness and kurtosis test for normal distribution. Indices smaller than 2 were considered to be normal distributed (Vincent, 2005). ANOVA repeated-measures with Bonferroni post-hoc test was used to compare the three testing sessions and the percentage of training time in each zone for each of the 6 months for both TID models. Statistical differences were considered to be significant for *p* < 0.05. The relationship between different parameters was investigated with Pearson's correlation coefficient. For these correlation analyses, all three time points were included. Cohen's effect size (d) was calculated for the comparison of all tests with each other. The thresholds for small, moderate, and large effects were defined as 0.20, 0.50, and 0.80, respectively (Cohen, 1988; Wahl et al., 2014).

## Results

**Figure 1:**
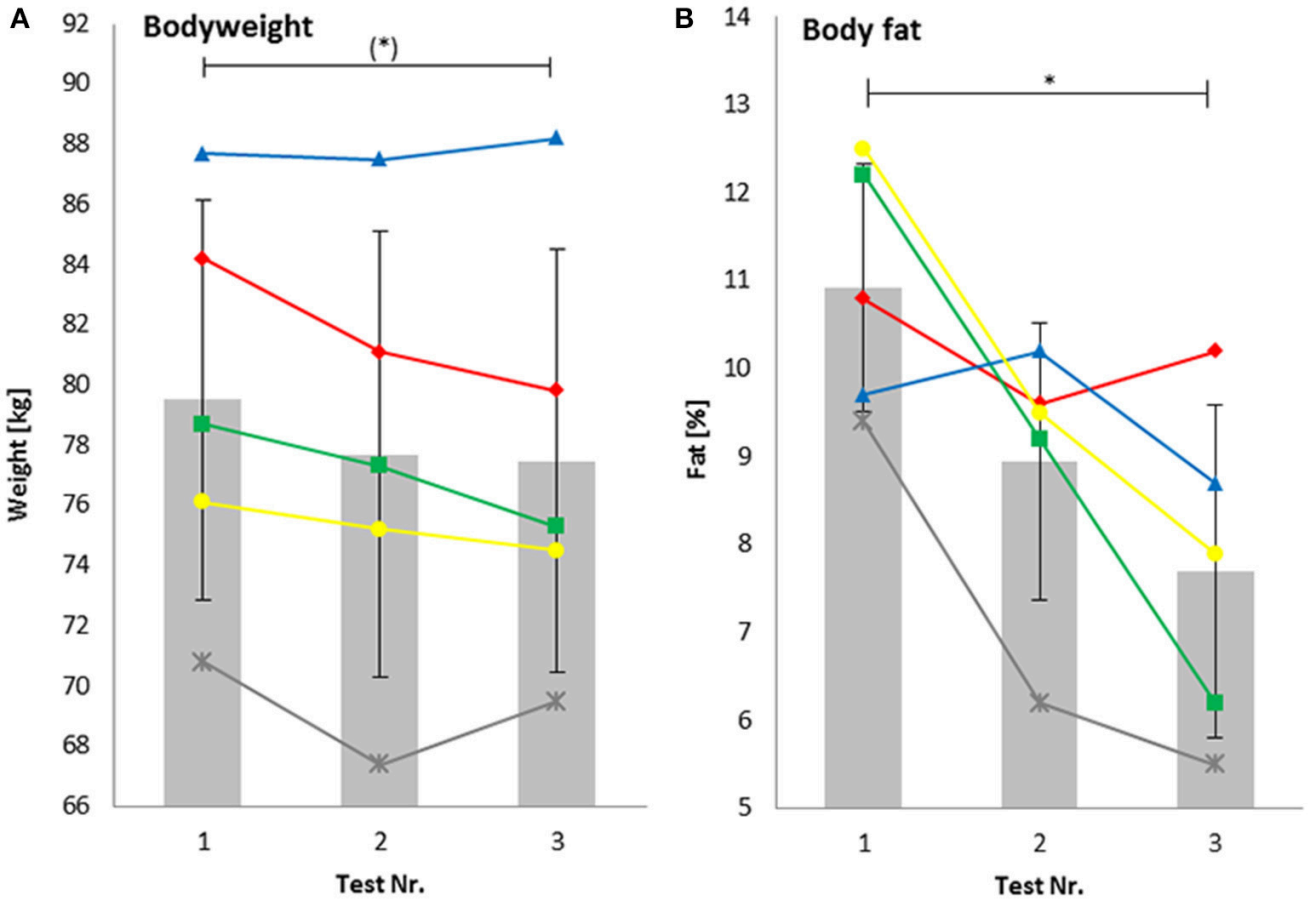
**Bodyweight (A)** body fat **(B)**. ^*^Significant difference; (^*^) values nearly reached statistical significance (*P* = 0.07).

The decrease in body mass during the total training period nearly reached statistical significance (−2.6 ± 2.3%, *P* = 0.07, *d* = 0.29). Body fat was significantly reduced by −28.7 ± 19.5% (*P* = 0.01, *d* = 1.91) as shown in Figures 1A,B.

### Strength-tests

There was no significant change in relative leg curl MVC (*P* = 1.0, *d* = 0.06), relative leg extension MVC (*P* = 1.0, *d* = 0.46) and leg press MVC (*P* = 1.0, *d* = 0.06). Jumping height in the CMJ (*P* = 1.0, *d* = 0.15) and in the SJ (*P* = 1.0, *d* = 0.16) changed not significantly (Table 2).

**Table 2:** **Maximal isometric strength (ISO MAX) and jump performance**.

	**Test Nr**.	**1**	**2**	**3**
ISO MAX	Leg Press [n·kg^−1^]	52.0 ± 12.1	43.9 ± 9.9	52.8 ± 17.0
	Leg Extension [n·kg^−1^]	28.5 ± 2.9	28.0 ± 3.0	29.84 ± 3.2
	Leg Curl [n·kg^−1^]	16.3 ± 1.0	16.5 ± 0.7	16.3 ± 1.2
Jumps	Squat Jump [cm]	31.68 ± 5.97	32.62 ± 7.58	32.02 ± 5.76
	CMJ [cm]	32.68 ± 6.43	34.04 ± 8.47	33.00 ± 6.89

### Sprint-test

The decreases in the relative PPO and MPO in the sprint nearly reached statistical significance [PPO: −3.2 ± 2.7%, *P* = 0.06, *d* = 0.25 (**Figure 3A**) and MPO: −3.6 ± 4.1%, *P* = 0.06, *d* = 0.45 (**Figure 3B**)].

dLa/dt_max_ decreased significantly by −16.3 ± 8.1% (*P* = 0.03, *d* = 0.73), relative dLa/dt_max_ kg^−1^ decreased significantly by −16.5 ± 6.7% (*P* = 0.02, *d* = 0.67) as shown in **Figure 3C**. A high correlation was found between relative MPO in the sprint and relative dLa/dt_max_ (*r* = 0.85, *P* < 0.001).

### VO_2max_-test

**Figure 2:**
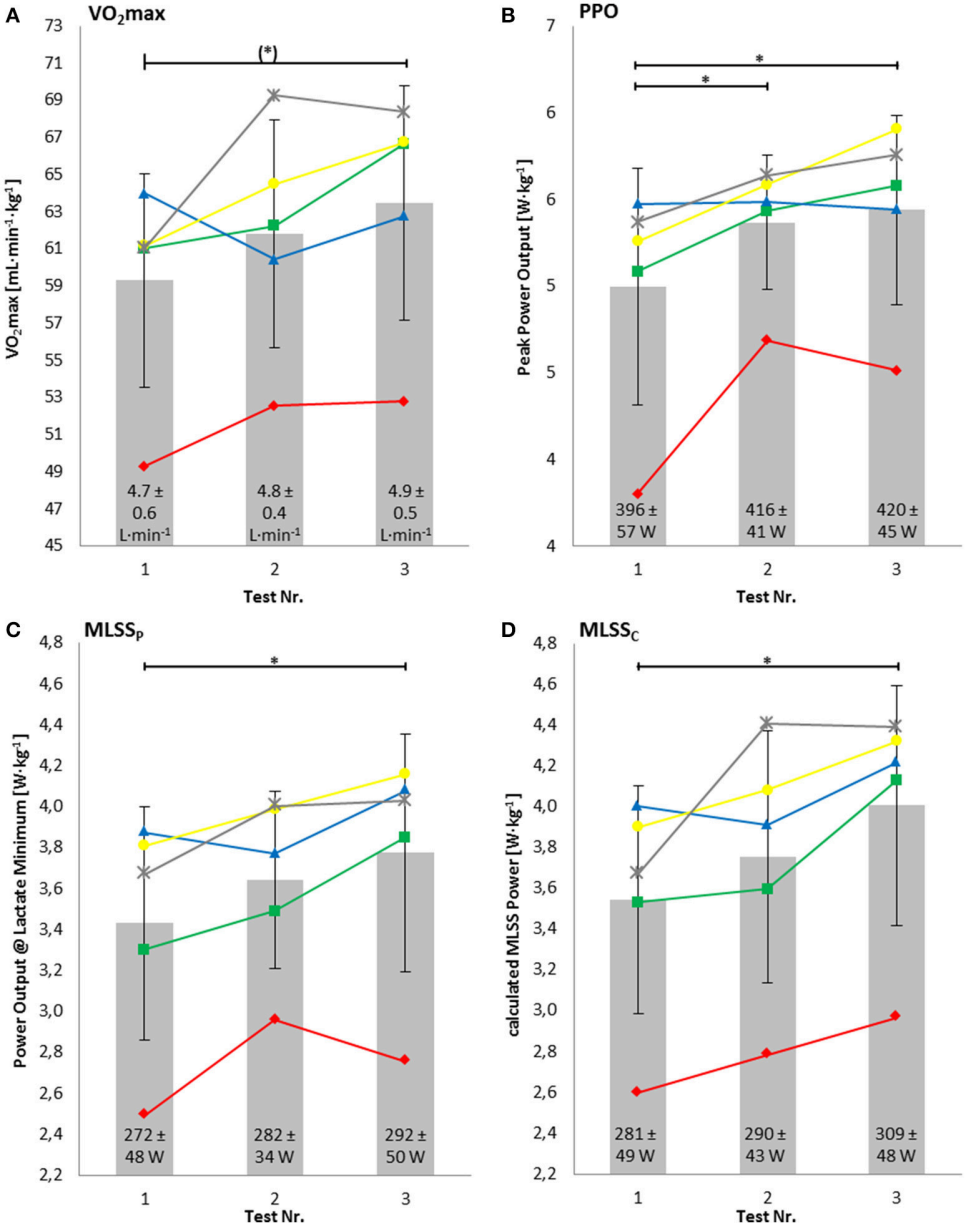
**Relative maximal oxygen uptake (VO_2max_) (A)**, relative peak power output (PPO) **(B)**, relative power output at lactate minimum (MLSS_P_) **(C)**, and relative power output at calculated maximal lactate steady state (MLSS_*C*_) **(D)**. Numbers within the bars represent the absolute values. ^*^Significant difference; (^*^) values nearly reached statistical significance (*P* = 0.06).

**Figure 3:**
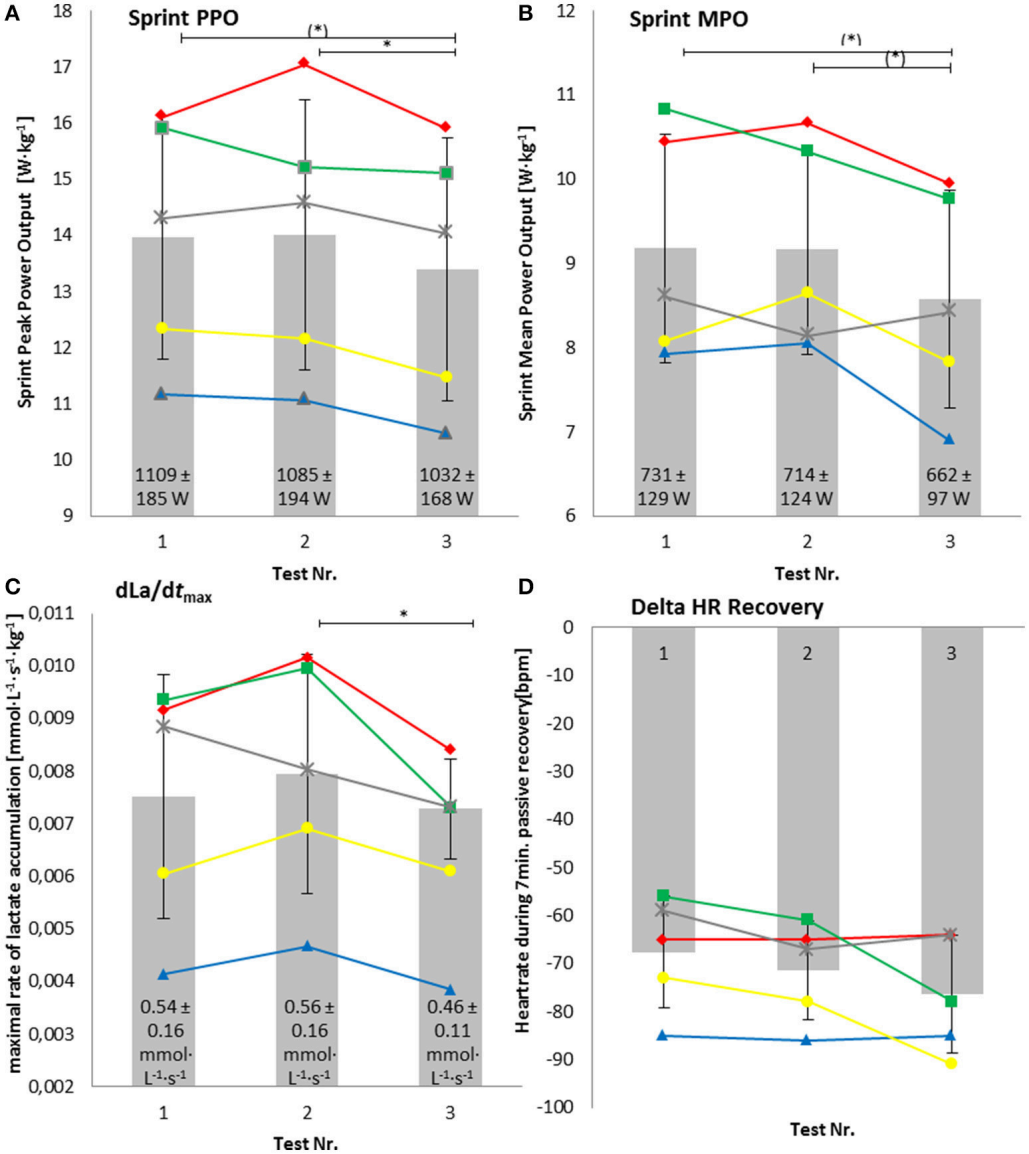
**Relative peak power output in the sprint test (Sprint PPO) (A)**, relative mean power output in the sprint test (Sprint MPO) **(B)**, maximal lactate production rate (dLa/dt_max_) **(C)**, and drop in heart rate after 7 min of passive recovery after maximal exhaustion (Delta HR Recovery) **(D)**. Numbers within the bars represent the absolute values. ^*^Significant difference; (^*^) values nearly reached statistical significance (*P* = 0.06).

The increase in relative VO_2max_ in the time course of the 6-month preparation phase nearly reached statistical significance (7.1 ± 5.3%, *P* = 0.06, *d* = 0.69) (Figure 2A). Relative peak power output in the ramp test significantly increased by 9.5 ± 7.1% (*P* = 0.02, *d* = 0.71) (Figure 2B). The drop in heart rate during 7 min of passive recovery showed no significant changes (−14.4 ± 17.2%, *P* = 0.13, *d* = 0.74) (Figure 3D), as well as peak heart rate in the ramp test (174 ± 10 to 173 ± 9 bpm).

### Lactate-minimum-test (LMT)

The relative MLSS_P_ increased significantly by 10.3 ± 4.1% (*P* = 0.01, *d* = 0.61) similar to the relative MLSS_c_, which increased significantly by 13.3 ± 5.5% (*P* = 0.008, *d* = 1.56) (Figures 2C,D). There was a high correlation of relative MLSS_c_ and the relative power output at MLSS_P_ (*r* = 0.94, *P* < 0.001). Relative MLSS_P_ showed a high positive correlation with relative VO_2max_ (*r* = 0.92, *P* < 0.001) and a negative correlation with absolute dLa/dt_max_ (*r* = −0.78, *P* < 0.001).

### Short power-profile

**Figure 4:**
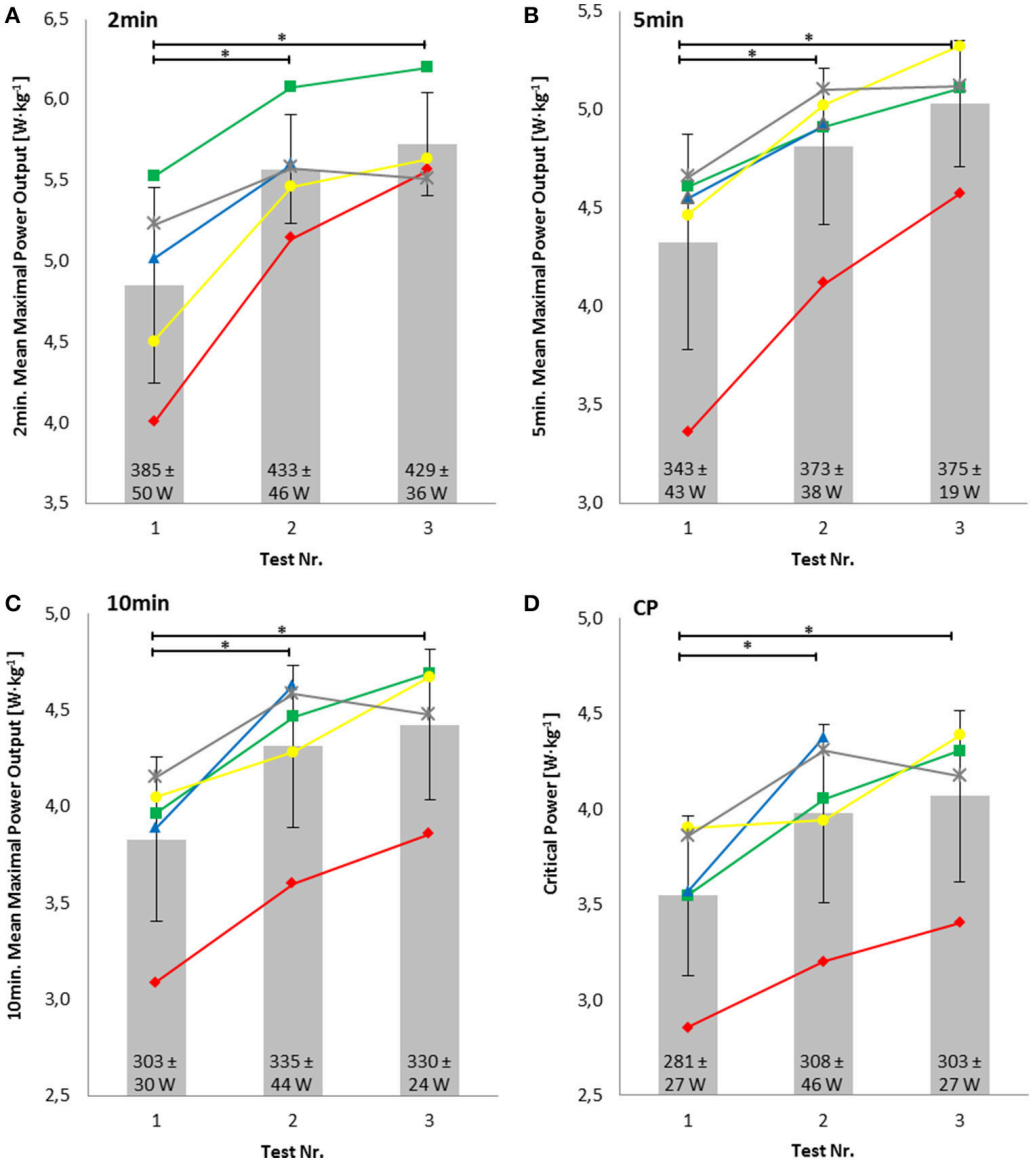
**Relative mean maximal power output in the field tests over 2 min (A)**, 5 min **(B)** and 10 min **(C)** and the calculated relative critical power (CP) **(D)**. Numbers within the bars represent the absolute values. ^*^Significant difference.

Relative MMP tested in the field over a duration of 2 min increased significantly by 18.7 ± 13.4% (*P* = 0.01, *d* = 1.81), 5 min increased significantly by 16.9 ± 11.6% (*P* = 0.002 *d* = 1.56), and 10 min increased significantly by 17.1 ± 6.3% (*P* = 0.001, *d* = 1.44) during the training phase. The calculated relative CP increased significantly by 16.8 ± 6.2% (*P* = 0.007, *d* = 1.20) (Figures 4A–D). Relative CP showed a high positive correlation with relative MLSS_P_ (*r* = 0.86, *P* < 0.001), relative MLSS_C_ (*r* = 0.88, *P* < 0.001), and relative VO_2max_ (*r* = 0.85, *P* < 0.001). Relative CP and dLa/dt_max_ showed a negative correlation of *r* = −0.61 (*P* < 0.02).

### Training data

**Figure 5:**
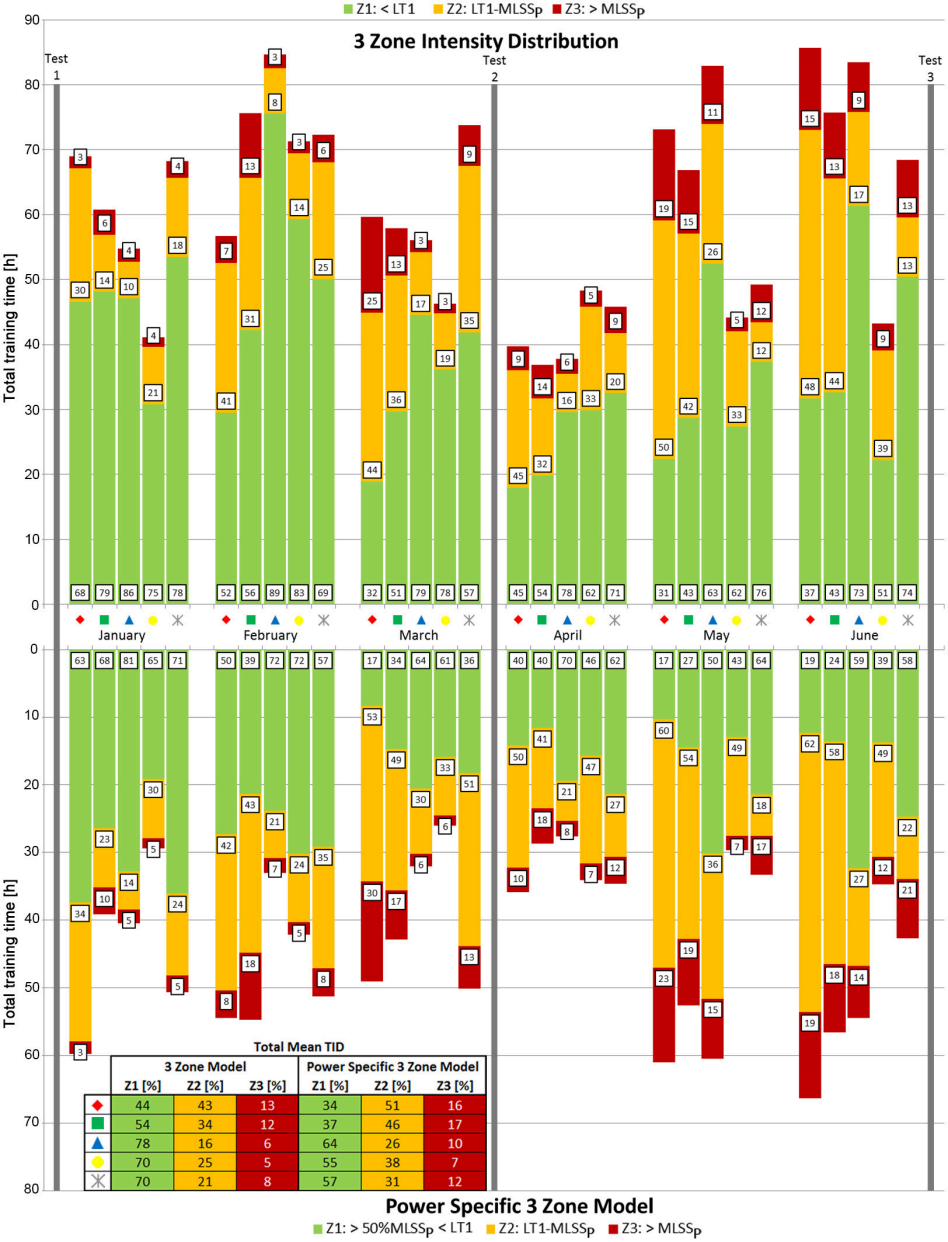
**Training intensity distribution according to the 3 zone model (top) and the power specific 3 zone model (bottom) over the time course of 6 months**. Numbers in boxes represent the percentage of total training time spent in each zone per month for each athlete. Vertical arrows assign performance tests. Symbols represent each individual athlete. The included table shows the TID of each athlete for the whole preparation period.

Total mean training time was 366 ± 41 h leading to a pyramidal TID (zone 1: 63 ± 16%, zone 2: 28 ± 13%, zone 3: 9 ± 5%) (Figure 5 top) and approximately ~15.3 h of training per week using the classical 3-zone model. Total mean training time without power output below 50% MLSS_P_ (power specific 3-zone model) was 261 ± 47 h and reflected a THR TID (zone 1: 48 ± 13%, zone 2: 39 ± 10%, zone 3: 13 ± 4%) (Figure 5 bottom). Overall-ANOVA revealed that the percentage of training in the HVT-zone was significantly higher (*P* = 0.003), the percentage of training in the THR-zone (*P* = 0.02) and HIT-zone (*P* = 0.008) was significantly lower in the classical 3-zone model compared to the power specific 3-zone model during each of the 6 months of training.

### Race data

Individual race data (time, distance, altitude difference, average relative power output, average power output, average power output in percent of MLSS_P_, average power output in percent of LT1, and average cadence) are shown in Table 3.

**Table 3:** **Competition data of each individual racer (colored symbols) during the Race Across America**.

**Athlete**	**Time [hh:mm]**	**Distance [km]**	**Altitude difference [m]**	**Average power [W·kg^−1^]**	**Average power [W]**	**Power [% MLSS]**	**Power [% LT1]**	**Average cadence [rpm]**
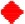	29:55	890	7411	2.3	186	85	141	78
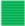	34:41	1081	9566	2.6	199	69	100	88
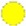	45:16	1628	9528	2.8	214	69	91	88
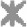	37:08	1184	9100	2.7	187	69	92	90

## Discussion

The aim of the present study was to analyze the TID for a Race Across America Team with two different approaches and to examine the corresponding development of physiological parameters as well as the actual performance over the course of the preparation for the race, where the team finished in second place. Over the training period of 6 months, the five athletes reduced their body fat significantly. Moderate increases were shown in relative VO_2max_ and moderate decreases in dLa/dt_max_. Power output at MLSS (MLSS_P_ and MLSS_C_) increased significantly together with performance as indicated by the calculated CP. As an additional indication of increased fitness levels, moderate effects were shown by the drop in heart rate within 7 min of passive recovery. The TID of the established 3-zone model reflected a PYR TID (zone 1: 63 ± 16%, zone2: 28 ± 13% and zone 3: 9 ± 4%). Deleting training time with power output below 50% of MLSS_P_, resulted in a THR TID (zone 1: 48 ± 13%, zone 2: 39 ± 10%, zone 3: 13 ± 4%).

The different results of our two approaches concerning TID, underline that the inherent variability in power output during training raises several challenges when attempting to evaluate the exact nature of a given training session. Different strategies on how to analyze power data have been suggested (Allen and Coggan, 2010). Allen and Coggan (2010) proposed using an exponentially weighted averaging process to represent the data. We decided to use an alternative approach, eliminating time with zero or very low power output, based on the assumption that a certain threshold intensity needs to be reached to result in an effective training stimulus (Davies and Knibbs, 1971). According to Meyer et al. (1999) and Wolpern et al. (2015), we decided to use a lactate threshold orientated lower fix point for the determination of zone 1 and set the minimum power output necessary to elicit a stimulus for training adaptation to 50% of MLSS_P_. Applying the classic 3-zone model in the present study, weekly training time is similar to a report of athletes participating in a RAAM qualifying race (Knechtle et al., 2012b). However, when using the power specific 3-zone model in our study, total training time is reduced by 104 ± 7 h per athlete, leading to a THR TID. Our athletes, therefore, performed a lot of training between LT1 and MLSS, which was shown to be a rather ineffective training intensity when the goals are to gain performance improvements and physiological adaptations (Esteve-Lanao et al., 2007; Yu et al., 2012; Stöggl and Sperlich, 2014).

Luciá et al. (2000) and Aagaard et al. (2011) studied professional cyclists and young top-level national cyclists for periods of seven (PYR TID) and four (unknown TID) months, respectively. Despite higher weekly training volumes compared to our athletes, they could not show significant changes in VO_2max_. The authors concluded that the very high fitness level of their athletes prevented further improvements for this parameter. In contrast, 7 months of a PYR TID with slightly more training per week (+ ~1 h) than above-mentioned studies, led to significant improvements in relative VO_2max_ of 10% in fourteen male young top-level national road cyclists (Zapico et al., 2007). In any way, lactate values for a given sub-maximal workload during a ramp test decreased after the 7 months of training, suggesting an increased reliance on oxidative metabolism (Luciá et al., 2000), MMP over 5 min increased significantly (Aagaard et al., 2011) and MLSS increased by 15% (Zapico et al., 2007), which is in line with our results. Since our cyclists were not top levels athletes, it is very unlikely that they reached a ceiling of improvement for VO_2max_. Nonetheless, there were only small increases in relative VO_2max_, which can mainly be attributed to reductions in body weight. It can be speculated that the amount of HIT/SIT, which was shown to be most effective in improving VO_2max_ (McKenna et al., 1997; Dawson et al., 1998; MacDougall et al., 1998; Breil et al., 2010; Gibala et al., 2012; Etxebarria et al., 2014; Milanović et al., 2015) was too low in the present study to increase VO_2max_ substantially, especially in the context of the limited regular training volume. However, the decrease in dLa/dt_max_ and the corresponding increase in MLSS_P_ power output underline the importance to document changes in these parameters, besides VO_2max_, in order to gain more insight into physiological changes, leading to performance improvements (e.g., the rise in CP).

Additionally, Zapico et al. (2007) observed parallel to an increase in PPO, a significant reduction in maximal heart rate during a ramp test after 7 months of training, which is in contrast to our athletes. It has to be taken into account, however, that the subjects in our study were ~25 years older and ~11 kg heavier, which might partly explain the overall lower values, especially in VO_2max_, as well as the smaller improvements. The TIDs, although being PYR in both studies, differ in so far, that our subjects trained fewer hours in total (−105 h) and to a less extent in zone 1 (−9.5%), and more in zone 2 (+6.5%). It can be speculated that the higher amount of THR training might be one reason for the lower gains in the physiological variables of the present study (Laursen, 2010). This assumption is supported by the study of Neal et al. (2011), who monitored 10 triathletes over a 6 month training period with a mean training volume of 203 ± 71 h. Similar to our athletes, these triathletes performed a PYR TID with a high amount of THR training, resulting in no changes in power output at lactate turn point. The lower effectiveness of a THR TID is further supported by another study of Neal et al. (2013), which resulted in greater improvements in 40-km time trial and LT1 and lactate turn point in a group of cyclists training POL when compared to a THR TID.

The LMT and the calculation of the MLSS (MLSS_C_) using VO_2max_ and dLa/dt_max_ were shown to be valid to determine MLSS (Knöpfli-Lenzin and Boutellier, 2011; Hauser et al., 2014a). The fact that both independent methods to determine the power output at MLSS showed a high correlation underlines that the increases in MLSS were accurately determined. Additionally, the present study is the first to document changes in MLSS over the course of a training period for cyclists using these two approaches. Only four previous studies investigated the effects of training on the lactate minimum. Carter et al. (1999) showed that the LMT is not sensitive to identify longitudinal effects of endurance training in sports students over 6 weeks, despite significant improvements in VO_2max_. Similar to our results, three other studies found significant increases in the LM intensity, investigating elite and youth soccer players (da Silva et al., 2007; Miranda et al., 2013) and youth swimmers (Campos et al., 2014), supporting that the LMT can be performed to identify longitudinal training effects. Again, this is further supported by the high correlation of the two methods we used to determine MLSS.

According to Hauser et al. (2014b), the MLSS is mainly influenced by VO_2max_ and dLa/dt_max_. While VO_2max_ and its influence on endurance performance has been focused on in most endurance studies (Coyle et al., 1988; Schumacher and Mueller, 2002; Støren et al., 2012), dLa/dt_max_ has been neglected and appears to be an underestimated parameter in terms of the origin and interpretation of MLSS (Mader and Heck, 1986).

The present study is the first to describe changes in the dLa/dt_max_ over a long training period, and also its correlation with performance (CP, MLSS, MPO). Based on the model of Mader and Heck (1986) only both together—the moderate increase in VO_2max_ (7.1%) and to a larger proportion the decrease in dLa/dt_max_(16.3%)—can explain the increase in performance (MLSS, CP) as measured in the present study. In accordance with the decrease in dLa/dt_max_ is the loss of anaerobic power, also reflected by the decrease in PPO and MPO within the sprint test. However, we found no significant changes in the strength assessments for five athletes. This is partly in line with the findings of Aagaard et al. (2011), where cyclists' maximal isometric strength, tested via leg press, also remained unchanged, after performing only endurance training over a period of 4 months.

The analysis of our race data showed, that three athletes used similar relative power outputs in relation to their MLSS_P_ and LT1. The race intensity of our athletes is in agreement with the data of Laursen et al. (1999), who described a mean intensity slightly below the ventilatory threshold of a 4 person RAAM team. A solo racer during the RAAM performed at slightly lower intensities (77% of LT1) (Schumacher et al., 2011). The weakest subject in our study performed more intense with regards to % of MLSS_P_ and % of LT1 during the race, which might be due to the lower distance and altitude differences that he had to cover compared to the other riders. Another explanation might be that this athlete had the highest relative amounts of training time in zone 2 and 3, compared to the stronger riders. It has been reported before, that the cycling speed during the training units was significantly and negatively related to race time (Knechtle et al., 2011, 2012b). Nevertheless, differences in race performances in ultra-endurance events can also result from various other influencing factors like race tactics, weather conditions, motivation (Lahart et al., 2013), sleep deprivation (Knechtle et al., 2012a), nutrition (Stewart and Stewart, 2007; Hulton et al., 2010; Bescós et al., 2012; Lahart et al., 2013; Paulin et al., 2015) and so on, which are not taken into account in this publication.

## Conclusion

The present study shows a THR orientated TID and only moderate (Cohen, 1988) increases in physiological parameters. The resulting TID, however, may largely depend on the kind of analysis of power data. The amount of training below 50% of MLSS in our amateur athletes is remarkably high (104 h (~28% of total training time)). Researchers, coaches, and athletes, either analyzing TID retrospectively or planning training in advance, should pay close attention to the different ways of interpreting training data, to gain realistic insights in TID and the corresponding improvements in performance and physiological parameters. In matters of physiological parameters determined during training periods, power output at LT1 seems to be a good estimate of long-term endurance performance. Future studies, therefore, should consider measuring dLa/dt_max_ and MLSS in addition to VO_2max_ and performance.

## Author contributions

CM: performed tests and statistics, acquired data, and wrote the paper. JM: wrote paper. WK: acquired data. PW: performed tests and statistics, acquired data, and wrote the paper.

### Conflict of interest statement

The authors declare that the research was conducted in the absence of any commercial or financial relationships that could be construed as a potential conflict of interest.

## References

[B1] Aagaard P. Andersen J. L. Bennekou M. Larsson B. Olesen J. L. Crameri R.. (2011). Effects of resistance training on endurance capacity and muscle fiber composition in young top-level cyclists. Scand. J. Med. Sci. Sports 21, e298–e307. 10.1111/j.1600-0838.2010.01283.x 21362056

[B2] Allen H. Coggan A. R. (2010). Training and Racing with a Power Meter. Boulder, CO: VeloPress.

[B3] Bagger M. Petersen P. H. Pedersen P. K. (2003). Biological variation in variables associated with exercise training. Int. J. Sports Med. 24, 433–440. 10.1055/s-2003-41180 12905092

[B4] Bescós R. Rodríguez F.-A. Iglesias X. Knechtle B. Benítez A. Marina M.. (2012). Nutritional behavior of cyclists during a 24-hour team relay race: a field study report. J. Int. Soc. Sports Nutr. 9:3. 10.1186/1550-2783-9-3 22309475 PMC3287968

[B5] Billat V. Demarle A. Slawinski J. Paiva M. Koralsztein J. P. (2001). Physical and training characteristics of top-class marathon runners. Med. Sci. Sports Exerc. 33, 2089–2097. 11740304 10.1097/00005768-200112000-00018

[B6] Bowen R. L. Adams J. H. Myburgh K. H. (2006). Nausea and high serum osmolality during a simulated ultraendurance adventure race: a case-control study. Int. J. Sports Physiol. Perform. 1, 176–185. 10.1123/ijspp.1.2.176 19114751

[B7] Breil F. A. Weber S. N. Koller S. Hoppeler H. Vogt M. (2010). Block training periodization in alpine skiing: effects of 11-day HIT on VO_2max_ and performance. Eur. J. Appl. Physiol. 109, 1077–1086. 10.1007/s00421-010-1455-1 20364385

[B8] Brown L. E. Weir J. P. (2001). ASEP procedures recommendation I: accurate assessment of muscular strength and power. J. Exerc. Physiol. 4, 1–21.

[B9] Buchheit M. (2014). Monitoring training status with HR measures: do all roads lead to Rome? Front. Physiol. 5:73. 10.3389/fphys.2014.00073 24578692 PMC3936188

[B10] Campos E. Z. Nordsborg N. B. da Silva A. S. R. Zagatto A. M. Gerosa Neto J. Andrade V. L. D. (2014). The response of the lactate minimum test to a 12-week swimming training. Motriz. Rev. Educ. Fis. 20, 286–291. 10.1590/S1980-65742014000300007

[B11] Carter H. Jones A. M. Doust J. H. (1999). Effect of 6 weeks of endurance training on the lactate minimum speed. J. Sports Sci. 17, 957–967. 10.1080/026404199365353 10622356

[B12] Cohen J. (1988). Statistical Power Analysis for the Behavioral Sciences. Hillsdale, NJ: Erlbaum.

[B13] Coyle E. F. Coggan A. R. Hopper M. K. Walters T. J. (1988). Determinants of endurance in well-trained cyclists. J. Appl. Physiol. 64, 2622–2630. 3403447 10.1152/jappl.1988.64.6.2622

[B14] da Silva A. S. R. Bonette A. L. Santhiago V. Gobatto C. A. (2007). Effect of soccer training on the running speed and the blood lactate concentration at the lactate minimum test. Biol. Sport 24, 105–114.

[B15] Davies C. T. Knibbs A. V. (1971). The training stimulus. The effects of intensity, duration and frequency of effort on maximum aerobic power output. Int. Z. Angew. Physiol. 29, 299–305. 5144913

[B16] Dawson B. Fitzsimons M. Green S. Goodman C. Carey M. Cole K. (1998). Changes in performance, muscle metabolites, enzymes and fibre types after short sprint training. Eur. J. Appl. Physiol. Occup. Physiol. 78, 163–169. 10.1007/s004210050402 9694316

[B17] Esteve-Lanao J. Foster C. Seiler K. S. Luci,á A. (2007). Impact of training intensity distribution on performance in endurance athletes. J. Strength Cond. Res. 21, 943–949. 10.1519/R-19725.1 17685689

[B18] Esteve-Lanao J. San Juan A. F. Earnest C. P. Foster C. Luciá A. (2005). How do endurance runners actually train? Relationship with competition performance. Med. Sci. Sports Exerc. 37, 496–504. 10.1249/01.MSS.0000155393.78744.86 15741850

[B19] Etxebarria N. Anson J. M. Pyne D. B. Ferguson R. A. (2014). High intensity cycle interval training improves cycling and running performance in triathletes. Eur. J. Sport Sci. 14, 521–529. 10.1080/17461391.2013.853841 24206175

[B20] Faria E. W. Parker D. L. Faria I. E. (2005). The science of cycling: physiology and training – part 1. Sports Med. 35, 285–312. 10.2165/00007256-200535040-00002 15831059

[B21] Gibala M. J. Little J. P. Macdonald M. J. Hawley J. A. (2012). Physiological adaptations to low-volume, high-intensity interval training in health and disease. J. Physiol. 590, 1077–1084. 10.1113/jphysiol.2011.224725 22289907 PMC3381816

[B22] Guellich A. Seiler K. S. Emrich E. (2009). Training methods and intensity distribution of young world-class rowers. Int. J. Sports Physiol. Perform. 4, 448–460. 10.1123/ijspp.4.4.448 20029096

[B23] Halson S. L. (2014). Monitoring training load to understand fatigue in athletes. Sports Med. 44(Suppl. 2), 139–147. 10.1007/s40279-014-0253-z 25200666 PMC4213373

[B24] Hauser T. Adam J. Schulz H. (2014a). Comparison of calculated and experimental power in maximal lactate-steady state during cycling. Theor. Biol. Med. Model. 11, 1–12. 10.1186/1742-4682-11-25 24886168 PMC4052616

[B25] Hauser T. Adam J. Schulz H. (2014b). Comparison of selected lactate threshold parameters with maximal lactate steady state in cycling. Int. J. Sports Med. 35, 517–521. 10.1055/s-0033-1353176 24227122

[B26] Heck H. Schulz H. Bartmus U. (2003). Diagnostics of anaerobic power and capacity. Eur. J. Sport Sci. 3, 1–23. 10.1080/17461390300073302

[B27] Hulton A. T. Lahart I. M. Williams K. L. Godfrey R. Charlesworth S. Wilson M.. (2010). Energy expenditure in the Race Across America (RAAM). Int. J. Sports Med. 31, 463–467. 10.1055/s-0030-1251992 20455193

[B28] Ice R. G. Millman P. L. Ice D. C. Camp J. C. (1988). A physiological profile of the 1984–1986 race across America winner, in Medical and Scientific Aspects of Cycling, eds Burke E. R. Newsom M. M.(Champaign, IL: Human Kinetics), 173–180.

[B29] Jacobs R. A. Rasmussen P. Siebenmann C. Díaz V. Gassmann M. Pesta D.. (2011). Determinants of time trial performance and maximal incremental exercise in highly trained endurance athletes. J. Appl. Physiol. 111, 1422–1430. 10.1152/japplphysiol.00625.2011 21885805

[B30] Knechtle B. Enggist A. Jehle T. (2005). Energy turnover at the Race Across AMerica (RAAM) – a case report. Int. J. Sports Med. 26, 499–503. 10.1055/s-2004-821136 16037895

[B31] Knechtle B. Knechtle P. Rust C. A. Rosemann T. Lepers R. (2011). Finishers and nonfinishers in the ‘Swiss Cycling Marathon’ to qualify for the ‘Race Across America’. J. Strength Cond. Res. 25, 3257–3263. 10.1519/JSC.0b013e31821606b3 22080313

[B32] Knechtle B. Wirth A. Knechtle P. Rüst C. A. Rosemann T. (2012b). A comparison of ultra-endurance cyclists in a qualifying ultra-cycling race for Paris-Brest-Paris and Race Across America-Swiss cycling marathon. Percept. Mot. Skills 114, 96–110. 10.2466/05.PMS.114.1.96-110 22582679

[B33] Knechtle B. Wirth A. Knechtle P. Rust C. A. Rosemann T. Lepers R. (2012a). No improvement in race performance by naps in male ultra-endurance cyclists in a 600-km ultra-cycling race. Chin. J. Physiol. 55, 125–133. 10.4077/CJP.2012.BAA022 22559737

[B34] Knöpfli-Lenzin C. Boutellier U. (2011). Lactate minimum is valid to estimate maximal lactate steady state in moderately and highly trained subjects. J. Strength Cond. Res. 25, 1355–1359. 10.1519/JSC.0b013e3181d6dbf4 21522075

[B35] Lahart I. M. Lane A. M. Hulton A. T. Williams K. L. Godfrey R. Pedlar C.. (2013). Challenges in maintaining emotion regulation in a sleep and energy deprived state induced by the 4800Km ultra-endurance bicycle race; the Race Across AMerica (RAAM). J. Sports Sci. Med. 12, 481–488. 24149155 PMC3772592

[B36] Laursen P. B. (2010). Training for intense exercise performance: high-intensity or high-volume training? Scand. J. Med. Sci. Sports 20(Suppl. 2), 1–10. 10.1111/j.1600-0838.2010.01184.x 20840557

[B37] Laursen P. B. Rhodes E. C. Buchanan J. M. (1999). Physiological analysis of a high intensity ultraendurance event. Strength Cond. J. 21, 26–38.

[B38] Luciá A. Hoyos J. Pardo J. Chicharro J. L. (2000). Metabolic and neuromuscular adaptations to endurance training in professional cyclists: a longitudinal study. Jpn. J. Physiol. 50, 381–388. 10.2170/jjphysiol.50.381 11016988

[B39] MacDougall J. D. Hicks A. L. MacDonald J. R. McKelvie R. S. Green H. J. Smith K. M. (1998). Muscle performance and enzymatic adaptations to sprint interval training. J. Appl. Physiol. 84, 2138–2142. 9609810 10.1152/jappl.1998.84.6.2138

[B40] Mader A. Heck H. (1986). A theory of the metabolic origin of “anaerobic threshold.” Int. J. Sports Med. 7(Suppl. 1), 45–65. 3744647

[B41] McKenna M. J. Heigenhauser G. J. McKelvie R. S. Obminski G. MacDougall J. D. Jones N. L. (1997). Enhanced pulmonary and active skeletal muscle gas exchange during intense exercise after sprint training in men. J. Physiol. 501(Pt 3), 703–716. 9218229 10.1111/j.1469-7793.1997.703bm.x PMC1159470

[B42] Meyer T. Gabriel H. H. Kindermann W. (1999). Is determination of exercise intensities as percentages of VO_2max_ or HRmax adequate? Med. Sci. Sports Exerc. 31, 1342–1345. 10487378 10.1097/00005768-199909000-00017

[B43] Milanović Z. Sporiš G. Weston M. (2015). Effectiveness of High-Intensity Interval Training (HIT) and continuous endurance training for VO_2max_ improvements: a systematic review and meta-analysis of controlled trials. Sports Med. 45, 1469–1481. 10.1007/s40279-015-0365-0 26243014

[B44] Miranda R. Antunes H. Pauli J. R. Puggina E. F. da Silva A. S. R. (2013). Effects of 10-week soccer training program on anthropometric, psychological, technical skills and specific performance parameters in youth soccer players. Sci. Sports 28, 81–87. 10.1016/j.scispo.2012.02.005

[B45] Monod H. Scherrer J. (1965). The work capacity of a synergic muscular group. Ergonomics 8, 329–338. 10.1080/00140136508930810

[B46] Muñoz I. Seiler K. S. Bautista J. España J. Larumbe E. Esteve-Lanao J. (2014). Does polarized training improve performance in recreational runners? Int. J. Sports Physiol. Perform. 9, 265–272. 10.1123/ijspp.2012-0350 23752040

[B47] Neal C. M. Hunter A. M. Brennan L. O'Sullivan A. Hamilton D. L. de Vito G.. (2013). Six weeks of a polarized training-intensity distribution leads to greater physiological and performance adaptations than a threshold model in trained cyclists. J. Appl. Physiol. 114, 461–471. 10.1152/japplphysiol.00652.2012 23264537

[B48] Neal C. M. Hunter A. M. Galloway S. D. (2011). A 6-month analysis of training-intensity distribution and physiological adaptation in Ironman triathletes. J. Sports Sci. 29, 1515–1523. 10.1080/02640414.2011.596217 21967604

[B49] Padilla S. Mujika I. Cuesta G. Goiriena J. J. (1999). Level ground and uphill cycling ability in professional road cycling. Med. Sci. Sports Exerc. 31, 878–885. 10378916 10.1097/00005768-199906000-00017

[B50] Paulin S. Roberts J. Roberts M. Davis I. (2015). A case study evaluation of competitors undertaking an antarctic ultra-endurance event: nutrition, hydration and body composition variables. Extrem. Physiol. Med. 4:3. 10.1186/s13728-015-0022-0 25767697 PMC4357189

[B51] Sandbakk Ø. Hegge A. M. Losnegard T. Skattebo Ø. Tønnessen E. Holmberg H.-C. (2016). The physiological capacity of the world's highest ranked female cross-country skiers. Med. Sci. Sports Exerc. 48, 1091–1100. 10.1249/MSS.0000000000000862 26741124 PMC5642331

[B52] Schumacher Y. O. Ahlgrim C. Prettin S. Pottgiesser T. (2011). Physiology, power output, and racing strategy of a Race Across America finisher. Med. Sci. Sports Exerc. 43, 885–889. 10.1249/MSS.0b013e3181fec009 20962691

[B53] Schumacher Y. O. Mueller P. (2002). The 4000-m team pursuit cycling world record: theoretical and practical aspects. Med. Sci. Sports Exerc. 34, 1029–1036. 10.1097/00005768-200206000-00020 12048333

[B54] Seiler K. S. Kjerland G. Ø. (2006). Quantifying training intensity distribution in elite endurance athletes: is there evidence for an “optimal” distribution? Scand. J. Med. Sci. Sports 16, 49–56. 10.1111/j.1600-0838.2004.00418.x 16430681

[B55] Stewart I. B. Stewart K. L. (2007). Energy balance during two days of continuous stationary cycling. J. Int. Soc. Sports Nutr. 4:15. 10.1186/1550-2783-4-15 17974033 PMC2164944

[B56] Stöggl T. L. Sperlich B. (2014). Polarized training has greater impact on key endurance variables than threshold, high intensity, or high volume training. Front. Physiol. 5:33. 10.1249/01.mss.0000486878.95285.a2 24550842 PMC3912323

[B57] Stöggl T. L. Sperlich B. (2015). The training intensity distribution among well-trained and elite endurance athletes. Front. Physiol. 6:295. 10.3389/fphys.2015.00295 26578968 PMC4621419

[B58] Støren Ø. Bratland-Sanda S. Haave M. Helgerud J. (2012). Improved VO_2max_ and time trial performance with more high aerobic intensity interval training and reduced training volume: a case study on an elite national cyclist. J. Strength Cond. Res. 26, 2705–2711. 10.1519/JSC.0b013e318241deec 22124353

[B59] Støren Ø. Ulevåg K. Larsen M. H. Støa E. M. Helgerud J. (2013). Physiological determinants of the cycling time trial. J. Strength Cond. Res. 27, 2366–2373. 10.1519/JSC.0b013e31827f5427 23238091

[B60] Tønnessen E. Sylta Ø. Haugen T. A. Hem E. Svendsen I. S. Seiler K. S. (2014). The road to gold: training and peaking characteristics in the year prior to a gold medal endurance performance. PLoS ONE 9:e101796. 10.1371/journal.pone.0101796 25019608 PMC4096917

[B61] Vincent W. J. (2005). Statistics in Kinesiology. Champaign, IL: Human Kinetics.

[B62] Wahl P. Güldner M. Mester J. (2014). Effects and sustainability of a 13-day high-intensity shock microcycle in soccer. J. Sports Sci. Med. 13, 259–265. 24790477 PMC3990877

[B63] Wahl P. Sanno M. Ellenberg K. Frick H. Böhm E. Haiduck B.. (2016). Aqua cycling does not affect recovery of performance, damage markers and sensation of pain. J. Strength Cond. Res. 10.1519/JSC.0000000000001462. [Epub ahead of print]. 27135478

[B64] Wolpern A. E. Burgos D. J. Janot J. M. Dalleck L. C. (2015). Is a threshold-based model a superior method to the relative percent concept for establishing individual exercise intensity? A randomized controlled trial. BMC Sports Sci. Med. Rehabil. 7:16. 10.1186/s13102-015-0011-z 26146564 PMC4491229

[B65] Yu H. Chen X. Zhu W. Cao C. (2012). A quasi-experimental study of Chinese top-level speed skaters' training load: threshold versus polarized model. Int. J. Sports Physiol. Perform. 7, 103–112. 10.1123/ijspp.7.2.103 22634959

[B66] Zapico A. G. Calderón F. J. Benito P. J. González C. B. Parisi A. Pigozzi F.. (2007). Evolution of physiological and haematological parameters with training load in elite male road cyclists: a longitudinal study. J. Sports Med. Phys. Fitness 47, 191–196. 17557057

